# Anti‐PD1 versus anti‐PD‐L1 immunotherapy in first‐line therapy for advanced non‐small cell lung cancer: A systematic review and meta‐analysis

**DOI:** 10.1111/1759-7714.13867

**Published:** 2021-02-14

**Authors:** Angelo Borsarelli Carvalho Brito, Marcos Pedro Guedes Camandaroba, Vladmir Cláudio Cordeiro de Lima

**Affiliations:** ^1^ Department of Medical Oncology A.C. Camargo Cancer Center São Paulo Brazil

**Keywords:** anti‐PD1, anti‐PD‐L1, immunotherapy, meta‐analysis, NSCLC

## Abstract

**Background:**

Due to the increasing number of trials with immune checkpoint inhibitors (ICIs) in the first‐line therapy of non‐small cell lung cancer (NSCLC) patients, we performed a systematic review and meta‐analyses to investigate the difference between anti PD‐1 and PD‐L1 antibodies, used alone or in combination with chemotherapy, through adjusted indirect analysis to minimize the potential bias regarding overall survival (OS), progression‐free survival (PFS), overall response rate (ORR) and grade 3–5 adverse events (AEs).

**Methods:**

A systematic review of studies reporting clinical outcomes and toxicity associated with first‐line therapy employing anti‐PD1 or anti‐PD‐L1 antibodies alone, or in combination with chemotherapy, to treat metastatic, treatment‐naïve NSCLC patients was performed. Primary outcomes were OS, PFS, ORR and grade 3–5 AEs. We used a random‐effects model to generate pooled estimates for proportions. Meta‐analyses using pooled risk ratios were performed for binary outcomes from comparative studies with the random effects model.

**Results:**

A total of 13 eligible studies met our eligibility criteria, including 7673 patients. In the ICI‐chemotherapy combination subgroup, we observed that anti‐PD1 therapy was associated with better OS (*p* = 0.022) and PFS (*p* = 0.029) compared with anti‐PD‐L1 therapy. In the monotherapy subgroup, there was no statistical difference between the use of anti‐PD‐1 and anti‐PD‐L1 for OS and PFS. With regard to ORR and toxicity, in the ICI‐chemotherapy combination subgroup, we observed a trend of better ORR (*p* = 0.12) with the use of anti‐PD1 therapy and less frequent grade 3–5 AEs compared to the use of anti‐PD‐L1 therapy (*p* = 0.0302). In the monotherapy subgroup, there was no statistical difference between the use of anti‐PD‐1 and anti‐PD‐L1 regarding ORR and toxicity.

**Conclusions:**

Our study suggests that PD‐1 drug plus chemotherapy is superior to anti‐PD‐L1 plus chemotherapy for NSCLC; nevertheless, as monotherapy, both strategies appear to be similar.

## Graphical abstract

Several immune checkpoint inhibitors (ICI) have been proven to play an important role in first‐line therapy of non‐small cell lung cancer (NSCLC); however, differences in performance and toxicity of anti‐PD‐1 and anti‐PD‐L1 drugs are seen in daily practice. The ICI‐chemotherapy combination subgroup was associated with better overall and progression‐free survival, a trend associated with a better response and less frequent toxicity. In the monotherapy subgroup, there was no statistical difference between the use of anti‐PD‐1 and anti‐PD‐L1 and prognosis, response rate or toxicity. The results of our meta‐analyses might impact on drug selection for clinical oncologists.

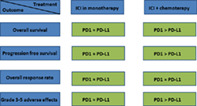

## INTRODUCTION

Immune checkpoint inhibitors (ICIs) have completely changed the treatment scenario and now represent an important therapeutic strategy for non–small cell lung cancer (NSCLC) patients. Specifically for NSCLC, anti‐PD1 or anti‐PD‐L1 antibodies are used building on the premise that by inhibiting the interaction between programmed cell death protein 1 (PD‐1) receptor expressed on activated T cells and PD‐L1 expressed in tumor cells that exhausted cytotoxic CD8+ T cells could be reinvigorated and elicit an effective antitumor response from the host's adaptive immune system.^1^ Currently, the first‐line therapy for NSCLC patients without actionable oncogenic drivers are ICIs; chemotherapy and antiangiogenic monoclonal antibodies (i.e., bevacizumab) can be used in association.

As first‐line therapy, the PD‐1 inhibitors, nivolumab and pembrolizumab, and the PD‐L1 inhibitors, atezolizumab and durvalumab, are good options and have been associated with improved response rate, improved survival and relatively low toxicity compared with chemotherapy.^2^, ^3^, ^4^, ^5^, ^6^, ^7^, ^8^, ^9^, ^10^, ^11^, ^12^, ^13^, ^14^

Due to the increasing number of trials with ICIs in NSCLC, potential differences between the performance and toxicity of anti‐PD‐1 and anti‐PD‐L1 drugs have attracted attention since this might impact drug selection. Therefore, we carried out a systematic review and meta‐analyses to address the differences between anti‐PD‐1 and anti‐PD‐L1 antibodies in first‐line therapy of metastatic NSCLC patients through adjusted indirect comparison to minimize the potential bias regarding overall response rate (ORR), progression‐free survival (PFS), overall survival (OS) and toxicity grade 3–5.

## METHODS

### Study design

A systematic review of studies that reported clinical outcomes and toxicity associated with first‐line therapy employing anti‐PD1 or anti‐PD‐L1 antibodies alone or in combination with chemotherapy to treat metastatic, treatment‐naïve NSCLC patients was performed.

### Search strategy

The search for eligible studies was performed in Embase, Medline and Cochrane Library, all from inception until May 30, 2019. Only data published in English was used. Applicable terms, such as “carcinoma, non‐small‐cell lung” OR “non‐small cell lung cancer” OR “NSCLC” AND “PD‐1” OR “PD‐L1” OR “programmed death receptor 1” OR “immune checkpoint inhibitor” OR “nivolumab” OR “pembrolizumab” OR “atezolizumab” OR “durvalumab” were used with the filter “clinical trial” when searching the PubMed‐MEDLINE, Embase and Scopus databases. Relevant abstracts were manually searched and retrieved from conference proceedings of the annual meetings of the American Society of Clinical Oncology, the International Association for the Study of Lung Cancer World Conference on Lung Cancer and the European Society for Medical Oncology.

The meta‐analysis included phase II or III randomized clinical trials (RCTs) in which the intervention arm employed ICIs (PD‐1 or PD‐L1 inhibitors) in monotherapy, or in combination with another chemotherapeutic agents. The target population consisted of previously untreated advanced/metastatic NSCLC patients. We excluded cohort studies, case–control studies, phase I trials or studies in which the outcomes measures could not be extracted from the published data. Trials involving pretreated patients were also excluded.

### Data collection

Two investigators (ACBC and MPGC) developed the search strategy and defined the eligible studies. From each study, two investigators (ACBC and MPGC) independently collected clinical information (ORR, PFS, OS, and grade 3–5 toxicity, according to therapy [ICI vs. non‐ICI therapy]). They compared their data to control for errors and a third investigator (VCCL) was consulted in case of discrepancies. The variables were defined a priori through a dictionary of word meaning.

This systematic review was in accordance with the Preferred Reporting Items for Systematic Review and Meta‐Analyses (PRISMA) statement.

### Statistical analysis

Descriptive statistics were used to summarize the characteristics of eligible studies. We performed meta‐analyses using pooled risk ratios for binary outcomes from comparative studies applying the random effects model. This model was chosen because despite the fact that we selected studies (randomized phase II and III trials) with robust results, some characteristics varied across studies, such as the percentage of different histologies, the rate of PD‐L1 expression in tumors, the immunohistochemical assessment of PD‐L1, among others. In the case of trials that included one or more investigational arms (CheckMate‐227, MYSTIC), the arm comparing ICI combination (for example, anti‐PD1 + anti‐CTLA4) was excluded from the analysis, and the comparison proceeded only between the control group (chemotherapy) and the ICI monotherapy (CheckMate‐227: nivolumab vs. chemotherapy; MYSTIC: durvalumab vs. chemotherapy) arm. In order to evaluate the presence of publication bias in comparative studies, we used Begg's funnel plot.^15^, ^16^

Since there have been no studies directly comparing PD1 and PD‐L1 treatments, we carried out indirect data analysis using the Excel spreadsheet developed by Catalá‐López et al., which can be accessed at http://metaanalisisenred.weebly.com/excel.html.^17^ Through this method, if it is necessary to compare treatment B versus treatment C in the absence of studies directly comparing B and C, one can use the effectiveness (effect size θ) of treatment B in relation to treatment A (θAB direct) and treatment C with the same comparator A (θAC direct), to perform an indirect comparison and obtain an estimate of the effect size between B and C (θBC indirect), designated as adjusted indirect comparison. Thus, it is possible to estimate effects of different interventions in systematic reviews even if there are no head‐to‐head comparisons between them. The findings of indirect comparisons and network meta‐analysis, nevertheless, allow for less certainty in conclusions than the findings of appropriate pairwise meta‐analyses of head‐to‐head trials.^18^

An I^2^ value of less than 50%, was considered significant when assessing heterogeneity across trials, i.e., low probability of result heterogeneity across studies.

All analyses were performed by REVMAN version 5.0. A two‐tailed *p*‐value of less than 0.05 was considered statistically significant.

## RESULTS

**FIGURE 1:**
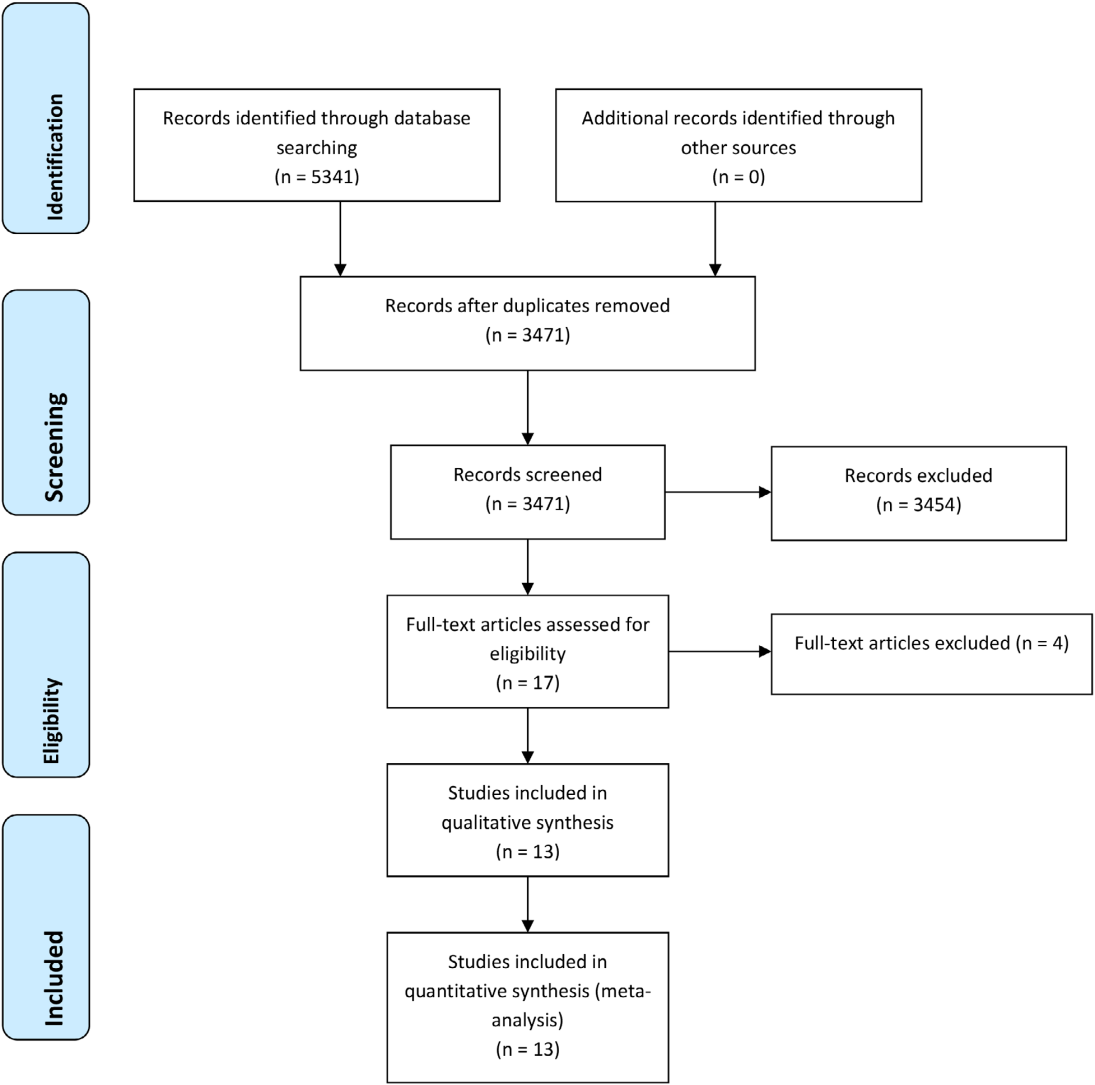
Flow chart depicting the selection algorithm and screening process

A total of 5341 studies were identified from the literature search, of which 3471 studies were excluded because of duplication. A total of 3454 studies were excluded after reviewing the titles and abstracts and 17 full‐text studies were further assessed for eligibility. In a second analysis, we excluded four studies. Finally, 13 eligible studies met our eligibility criteria. Figure 1 show the 13 RCTs included in the final analysis.

A total of 7673 patients were analyzed, of which 4077 patients were in the experimental group and 3596 patients in the control group. The majority of trials were phase III (12/13), except for one randomized controlled phase II trial (KEYNOTE 021). Table 1 summarizes treatment outcomes each study included in the present study.

**TABLE 1:** Summary of treatment outcomes of the studies included in this review and meta‐analysis

Study	Ref	Year	Study group			Control group				
			*N*	ORR (%)	Toxicity G3‐5 (%)	*N*	ORR (%)	Toxicity G3‐5 (%)	PFS HR IC 95%	OS HR IC 95%
ICI in monotherapy										
KEYNOTE 024	3	2016	154	44.8	26.6	151	27.8	53.3	0.50 (0.37–0.68)	0.60 (0.41–0.89)
CHECKMATE 026	4	2017	271	26.0	18.0	270	33.0	51.0	1.19 (0.97–1.46)	1.08 (0.87–1.34)
CHECKMATE 227	9	2019	396	35.9	32.8	397	30.0	36.0	0.82 (0.69–0.97)	0.79 (0.65–0.96)
KEYNOTE 042	10	2019	637	27.0	18.0	637	27.0	41.0	1.07 (0·94–1·21)	0.81 (0.71–0.93)
MYSTIC	13	2020	163	35.6	14.9	162	37.7	33.8	0.87 (0.59–1.29)	0.76 (0.56–1.02)
IMPOWER 110	14	2020	277	29.2	31.8	277	31.8	53.6	0.77 (0.63–0.94)	0.83 (0.65–1.07)
ICI in combination with chemotherapy										
KEYNOTE 021	2	2016	60	55.0	39.0	63	29.0	26.0	0.53 (0.31–0.91)	0.90 (0.42–1.91)
IMPOWER 132	5	2018	292	47.0	69.0	286	32.0	59.0	0.60 (0.49–0.72)	0.81 (0.64–1.03)
KEYNOTE 189	6	2018	410	47.6	67.2	206	18.9	65.8	0.52 (0.43–0.64)	0.49 (0.38–0.64)
KEYNOTE 407	7	2018	278	57.9	69.8	281	38.4	68.2	0.56 (0.45–0.70)	0.64 (0.49–0.85)
IMPOWER 150	8	2018	356	63.5	58.5	336	48.0	50.0	0.62 (0.52–0.74)	0.78 (0.64–0.96)
IMPOWER 130	11	2019	451	49.2	75.0	228	31.9	61.0	0.64 (0.54–0.77)	0.79 (0.64–0.98)
IMPOWER 131	12	2020	169	49.1	69.8	140	40.7	68.2	0.71 (0.60–0.85)	0.88 (0.73–1.05)

The studies showed some degree of heterogeneity regarding tumor histology (squamous vs. nonsquamous), chemotherapy protocol, distribution of ECOG performance status score, tobacco status and PD‐L1 expression. Trials evaluating ICI in monotherapy included all NSCLC histology. Among them, the percentage of squamous carcinoma varied from 18.8% (KEYNOTE 024) to 38.0% (KEYNOTE 042). Trials that tested the combination of ICI and chemotherapy included only squamous or nonsquamous patients.

Treatment chemotherapy protocols also varied across trials. It is important to note that despite including only nonsquamous patients, the IMpower 130 and IMpower 150 trials used taxanes instead of pemetrexed, as was used in the KEYNOTE 021 and KEYNOTE 189. Regarding performance status, the percentage of ECOG 0 patients in the monotherapy trials was lower than in the ICI and chemotherapy combination groups, with the exception of KEYNOTE 407 and IMpower 131.

Clinical characteristics, treatment arms and demographics from each study included in this meta‐analysis are described and summarized in Table S1 (online only).

Six trials investigated immunotherapy as monotherapy versus chemotherapy; four were with a PD1 inhibitor (one with anti CTLA‐4) and three with a PD‐L1 inhibitor. Seven trials investigated immunotherapy in combination with chemotherapy in first‐line; three investigated the anti‐PD1 inhibitor pembrolizumab and three investigated the anti‐PD‐L1 atezolizumab.

We did not include the comparison between nivolumab + chemotherapy versus chemotherapy from CheckMate‐227, because only patients whose tumors expressed PD‐L1 < 1% were enrolled in this part of the trial.

Standard chemotherapy (platin doublet) was only used as a control in all trials, except IMpower 150 that used chemotherapy + bevacizumab in both the control and investigational arms.

### Treatment outcomes: Survival endpoints

#### Overall survival

Regarding anti‐PD1 inhibitors, OS analysis was based on seven trials, from which four used ICI monotherapy and three used ICIs in combination with chemotherapy. The monotherapy group demonstrated a trend for better OS compared with the chemotherapy group (HR 0.86; 95% CI: 0.73–1.02), whereas the ICI‐chemotherapy combination was clearly associated with better OS (HR 0.59; 95% CI: 0.45–0.76) (Supplementary Online Figure S1).

For anti‐PD‐L1 inhibitors, OS analysis was based on six trials, from which two used ICI monotherapy and four used ICIs in combination with chemotherapy. Both the monotherapy and ICI‐chemotherapy combination groups were associated with better OS compared with chemotherapy (HR 0.80; 95% CI: 0.66–0.97 and HR 0.82; 95% CI 0.74–0.91, respectively) (Supplementary Online Figure S2).

**FIGURE 2:**
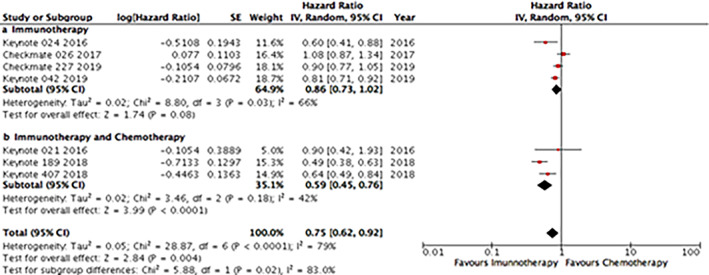
Indirect analysis statistical approach to compare overall survival (OS) between anti‐PD1 and anti‐PD‐L1 in monotherapy in (a) four and two studies, respectively, and (b) in combination with chemotherapy (three and four studies, respectively. In each line of the comparisons, A is the control group, B is the PD‐1 group and C is the PD‐L1 group

Through an indirect comparison, we analyzed the difference in terms of OS between anti‐PD1 versus anti‐PD‐L1 drugs (Figure 2). In the monotherapy group, there were no differences between both strategies (*p* = 0.57). Regarding the ICI‐chemotherapy combination group, anti‐PD1 antibodies were associated with better OS when compared with anti‐PD‐L1 drugs (*p* = 0.022).

#### Progression‐free survival

Regarding anti‐PD1 inhibitors, PFS analyses was based on seven trials. In four, ICI monotherapy was used and, in the other three, ICIs were used in combination with chemotherapy. While the monotherapy group was not statistically different when compared with the chemotherapy group (HR 0.91; 95% CI: 0.70–1.18), the ICI‐chemotherapy combination was associated with improved PFS (HR 0.54; 95% CI: 0.47–0.62) when compared with chemotherapy (Supplementary Online Figure S3).

For anti‐PD‐L1 inhibitors, PFS analysis was based on six trials, from which two used ICI monotherapy and four used ICI in combination with chemotherapy. In the monotherapy group, PFS associated with anti‐PD1 antibodies use as isolated drugs were not statistically different from that seen with chemotherapy (HR 0.86; 95% CI: 0.64–1.15), meanwhile, ICI‐chemotherapy combination was clearly associated with better PFS (HR 0.65; 95% CI: 0.59–0.71) (Supplementary Online Figure S4).

When we analyzed only patients whose tumors had PD‐L1 expression >50% (KEYNOTE‐024, KEYNOTE042 and IC3/TC3 [IMpower11]), ICI monotherapy was associated with better PFS (HR 0.69; 95% CI: 0.54–0.90) and better OS (HR 0.72; 95% CI: 0.61–0.85) (Supplementary Online Figure S5).

**FIGURE 3:**
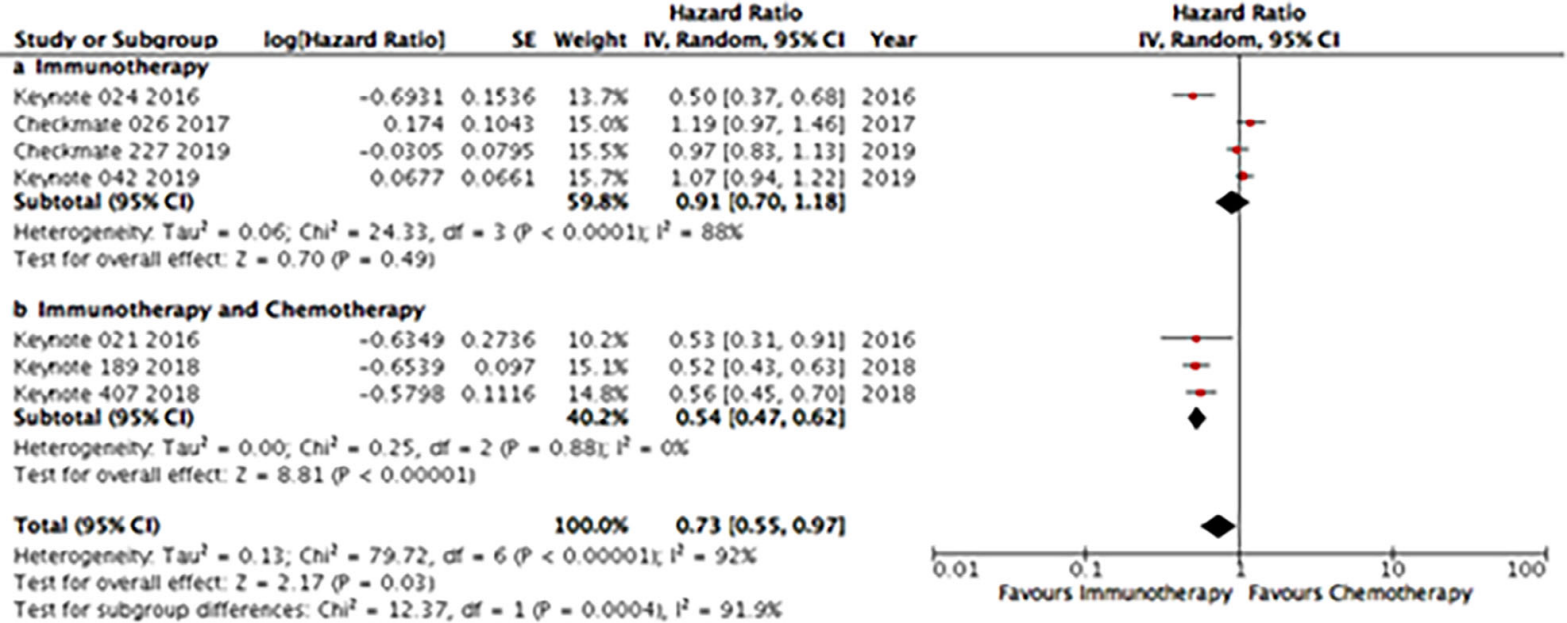
Indirect analysis statistical approach to compare progression‐free survival (PFS) between anti‐PD1 and anti‐PD‐L1 in monotherapy in (a) four and two studies, respectively, and (b) in combination with chemotherapy (three and four studies, respectively). In each line of the comparisons, A is the control group, B is the PD‐1 group and C is the PD‐L1 group

Through an indirect analysis, we tested the difference in terms of PFS between studies that evaluated anti‐PD1 versus anti‐PD‐L1 drugs (Figure 3). In the monotherapy group, there were no differences between the different immunotherapy strategies (*p* = 0.77). However, in the ICI‐chemotherapy combination group, anti‐PD1 drugs were associated with better PFS than anti‐PD‐L1 drugs as first‐line therapy for NSCLC (*p* = 0.029).

### Treatment outcomes: Overall response rate (ORR)

Regarding anti‐PD1 inhibitors, ORR analysis was based on seven trials, ICI monotherapy was used in four of them and in three of them ICIs were used in combination with chemotherapy. In the monotherapy group, there was no difference in ORR between patients treated with anti‐PD1 inhibitors and with chemotherapy (OR 1.02; 95% CI: 0.80–1.31), on the other hand, in the ICI‐chemotherapy combination group, anti‐PD1 drugs were associated with better ORR (OR 1.91; 95% CI: 1.34–2.71) (Supplementary Online Figure S6).

For anti‐PD‐L1 inhibitors, ORR was based on six trials, in which two of them used ICI monotherapy and four used ICIs in combination with chemotherapy. In the monotherapy group, there was no difference in ORR between treatment with anti‐PD1 inhibitors or chemotherapy (OR 0.93; 95% CI: 0.77–1.12), conversely, ICI‐chemotherapy combination was associated with improved ORR (OR 1.37; 95% CI 1.25–1.51) when compared with chemotherapy only (Supplementary Online Figure S7).

**FIGURE 4:**
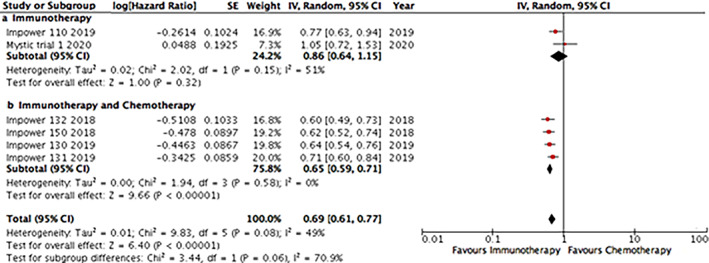
Indirect analysis statistical approach to compare overall response rate (ORR) between anti‐PD1 and anti‐PD‐L1 in monotherapy in (a) four and two studies, respectively, and (b) in combination with chemotherapy (three and four studies, respectively. In each line of the comparisons, A is the control group, B is the PD‐1 group and C is the PD‐L1 group

Through an indirect comparison, we analyzed the difference in terms of ORR between studies that uses anti‐PD1 or anti‐PD‐L1 drugs (Figure 4). In the monotherapy group, there was no difference between different immunotherapy strategies (*p* = 0.56). However, in the ICI‐chemotherapy combination group, anti‐PD1 was associated with a trend to better ORR compared with anti‐PD‐L1 as first‐line therapy for NSCLC (*p* = 0.12).

### Treatment outcomes: Grade 3–5 adverse events (AE)

Regarding anti‐PD1 inhibitors, the analysis of toxicity was based on seven trials, in four of them, ICI monotherapy was used, and in three ICIs were used in combination with chemotherapy. The monotherapy group was less associated with grade 3–5 AEs when compared with the chemotherapy group (OR 0.45; 95% CI: 0.38–0.54), while there was no difference between anti‐PD1 plus chemotherapy compared with chemotherapy only in the rate of grade 3–5 AE (OR 1.03; 95% CI: 0.95–1.12) (Supplementary Online Figure S8).

For anti‐PD‐L1 inhibitors, the analysis of the rate of grade 3–5 AEs was based on six trials; in two of them, ICI monotherapy was used and, in four of them, ICIs were used in combination with chemotherapy. There was less frequent grade 3–5 AEs with anti‐PD‐L1 drugs in the monotherapy group compared with the chemotherapy group (HR 0.54; 95% CI: 0.40–0.72), nonetheless, in the ICI‐chemotherapy combination studies evaluating anti‐PD‐L1 drugs were associated with more frequent grade 3–5 AEs compared with chemotherapy only (HR 1.16; 95% CI: 1.08–1.24) (Supplementary Online Figure S9).

**FIGURE 5:**
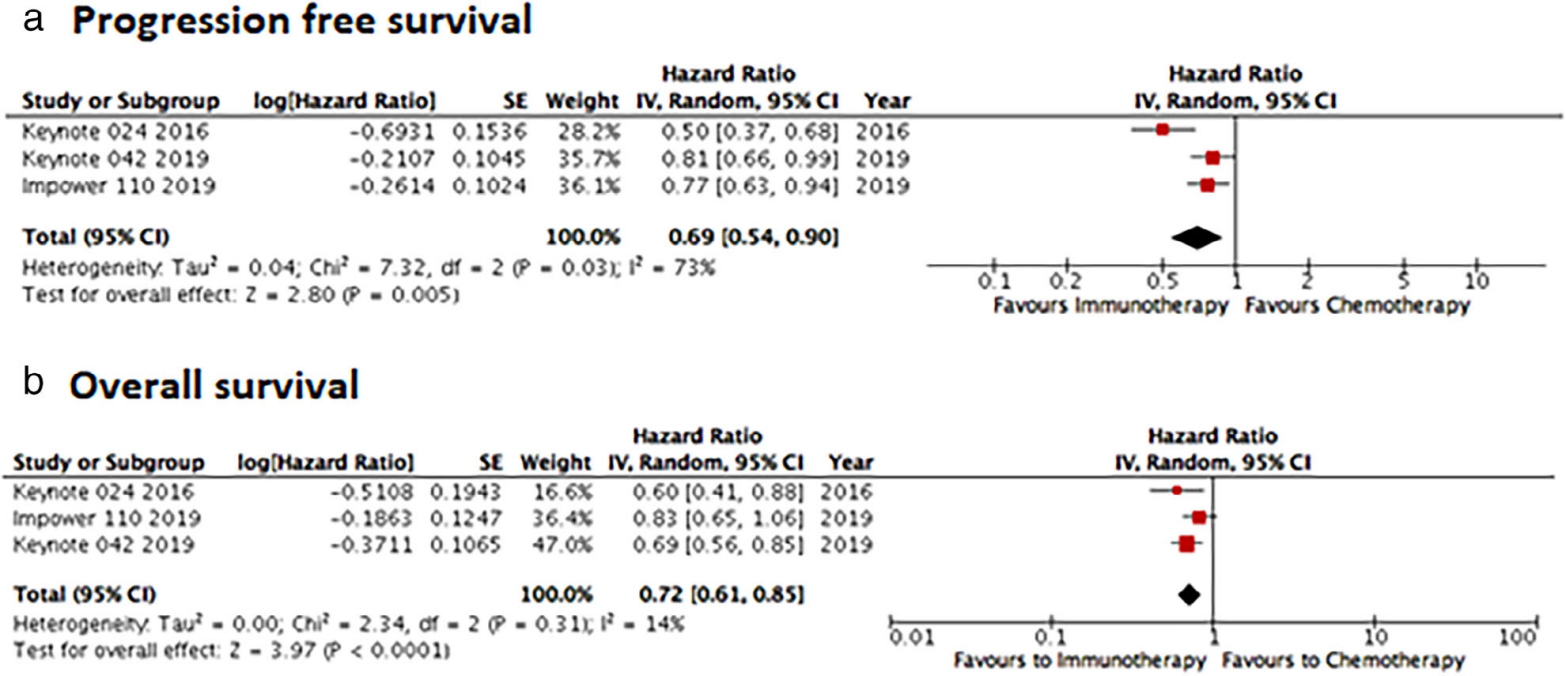
Indirect analysis statistical approach to compare the rate of grade 3–5 adverse events (AEs) between anti‐PD1 and anti‐PD‐L1 in monotherapy in (a) four and two studies, respectively, and (b) in combination with chemotherapy (three and four studies, respectively. In each line of the comparisons, A is the control group, B is the PD‐1 group and C is the PD‐L1 group

We indirectly compared the rate of grade 3–5 AEs between studies that used anti‐PD1 versus anti‐PD‐L1 drugs (Figure 5). In the monotherapy group, there were no differences between the different immunotherapy strategies (*p* = 0.32). In the ICI‐chemotherapy combination group, studies employing anti‐PD‐L1 drugs were associated with a higher rate of grade 3–5 AEs compared with anti‐PD1 drugs in first line for NSCLC (*p* = 0.0302).

## DISCUSSION

The use of ICIs with anti‐PD1 and anti‐PD‐L1 drugs has become the standard of care for patients with metastatic NSCLC without driver mutations in the first‐line setting. Although both groups of drugs have activity in NSCLC it is still unclear how these agents compare in terms of efficacy and toxicity. Therefore, this indirect meta‐analysis was conducted to analyze the efficacy and toxicity of anti‐PD‐1 and anti‐PD‐L1.

Through indirect analysis, we first demonstrated that the combination of anti‐PD1 and chemotherapy was associated with better OS and PFS when compared with anti‐PDL1 plus chemotherapy. Since IMpower 150 used bevacizumab, one can argue it might add a high degree of heterogeneity to the pooled data. However, the PRONOUNCE trial^19^ demonstrated that the schedule containing carboplatin plus paclitaxel and bevacizumab followed by maintenance with bevacizumab was in all aspects similar to carboplatin plus pemetrexed followed by maintenance with pemetrexed for nonsquamous NSCLC; thus, we decided to keep this trial in the analysis. In addition, bevacizumab was employed in both the control and investigational arms. The arm testing chemotherapy plus atezolizumab only was not included in the present meta‐analysis. In fact, we performed an additional analysis excluding the IMpower 150 trial. Results concerning OS, PFS and ORR remained the same as previously reported in Figures 3, 4, 5. With regard to toxicity, we observed a trend of higher rate of grade 3–5 AEs associated with anti‐PD‐L1 drugs when compared with anti‐PD1 drugs, although they were no longer statistically significant (Supplementary Online Figures S10–S13).

Second, we observed a trend of association of better ORR of anti‐PD1 drugs when compared with anti‐PD‐L1 in monotherapy. When we compared anti‐PD1 and anti‐PD‐L1 among high PD‐L1 expressors (patients whose tumors had TPS ≥ 50% or IC3/TC3), anti‐PD1 therapy was also associated with improved OS and PFS.

Together, these two results might imply that anti‐PD1 antibodies induce stronger antitumoral therapy.^20^ These differences might also derive from heterogeneous immunogenicity of the antibodies themselves, eliciting neutralizing antibodies against the anti‐PD1 or ant‐PD‐L1 antibodies that could dampen their activity to different extents.^21^

Finally, we observed a higher frequency of grade 3–5 AEs with anti‐PD‐L1 plus chemotherapy when compared with anti‐PD1 plus chemotherapy. Nonetheless, this may have resulted from the fact that taxane‐containing regimens were more frequently used in concert with anti‐PD‐L1 therapies, leading to more hematological and neurological toxicity when compared with pemetrexed‐containing protocols (Supplementary Online Table S1).

Since there are no published trials comparing anti‐PD‐1 and anti‐PD‐L1 in untreated metastatic NSCLC, the present data highlights that different ICIs might have distinct efficacy and toxicity in this population. There are few data addressing this issue in the literature.

Previous data from two large phase 1 studies, published in 2012, that included advanced cancers (NSCLC, melanoma, and renal cell cancer) testing the anti‐PD1 antibody, nivolumab (BMS‐936558), and an anti‐PD‐L1 antibody (BMS‐936559) demonstrated a higher overall response rate for the anti‐PD1 (20–25%) than for the anti‐PD‐L1 (6–17%).^22^, ^23^

A recently published meta‐analysis, led by a Chinese group, compared the OS differences between anti‐PD1 and anti‐PD‐L1 across different cancer types in 19 randomized clinical trials involving 11 379 patients, regardless of the number of previous treatments.^24^ They concluded that anti‐PD1 drugs were associated with better OS (HR 0.75; 95% CI: 0.65–0.86; *p* < 0.001) and PFS (HR 0.73; 95% CI: 0.56–0.96; *p* = 0.02) when compared with anti‐PD‐L1 drugs. No significant difference was observed in relation to the safety profile.

Two previous studies have suggested that anti‐PD1 drugs are associated with improved OS and PFS compared with anti‐PD‐L1 drugs in pretreated advanced NSCLC patients.^25^, ^26^ Focusing on toxicity, a systematic review involving 5744 patients concluded that the toxicity profile of PD‐1 and PD‐L1 inhibitors in NSCLC patients is similar.^27^

A hypothetical explanation for the improved efficacy of anti‐PD1 drugs compared with anti‐PD‐L1 antibodies could be that anti PD‐1 antibodies are able to inhibit the binding of PD‐1 not only to PD‐L1 but also to PD‐L2, which is an important interaction that also inhibits the activation of T cells.^28^ In fact, previous data described PD‐L2 as a potential predictive factor for ICI response. One study evaluated the expression of PD‐L2 in a mixed group of patients diagnosed with renal cell carcinoma (*N* = 59), melanoma (*N* = 38), metastatic urothelial carcinoma (*N* = 251), and NSCLC (*N* = 112). In this study, PD‐L2 expression was associated with improved OS following anti‐PD‐L1 therapy with atezolizumab.^29^ Another study evaluating recurrent or metastatic head and neck squamous cell carcinoma (HNSCC) patients found an association between PD‐L2 expression and clinical response to pembrolizumab.^30^

An important limitation of our study was related to the evaluation of PD‐L1 expression. We know that PD‐L1 expression is predictive of ICI response; however, once not all studies presented their results according to each category of PD‐L1 expression, these data could not be evaluated extensively. In fact, this data is not available for all studies included in this meta‐analysis. Another important point is the fact that trials which included only patients with high PD‐L1 expression might have influenced the results; nevertheless there is some heterogeneity in the expression level considered to classify tumors as high expressors; for example, the cutoff for PD‐L1 expression in KEYNOTE 024 and MYSTIC trials was 50% and 25%, respectively. It is also important to note that different studies utilized different assays to detect PD‐L1 expression. In the KEYNOTE studies, the 22C3 pharmDx assay was used to evaluate TPS (percentage of tumor cells with positive membranous PD‐L1 staining), while in the IMpower studies, the SP142 assay was used to detect PD‐L1 expression on tumor cells and tumor‐infiltrating immune cells. Thus, differences between these detection methods also confer heterogeneity in the comparison between trials.

Another important limitation of our study is the fact that we extracted the data related to survival (HRs and corresponding 95% CI), ORR and toxicity from the original published studies and did not have access to individual patient data. This prevented a thorough analysis of factors that may bring heterogeneity to the present analysis, such as the distribution of histological subtypes and the expression of PD‐L1 in tumors.

In conclusion, our meta‐analysis based on 13 studies including more than 7600 patients, indicates a slight superiority of anti‐PD1 antibodies over anti‐PD‐L1 inhibitors when used in combination with chemotherapy as first‐line therapy for metastatic NSCLC patients; as monotherapy, both ICI strategies appear to be similar.

## CONFLICT OF INTEREST

The authors confirm that there are no conflicts of interest.

## Supporting information


**Figure S1**. Pooled analysis of overall survival (OS) for anti‐PD1 drugs, alone or in combination, in first‐line therapy for non‐small carcinoma lung cancer (NSCLC) patients. (a) Studies with immunotherapy in monotherapy, and (b) studies with immunotherapy combined with chemotherapy
**Figure S2**. Pooled analysis of overall survival (OS) for anti‐PD‐L1drugs, alone or in combination, in first‐line therapy for non‐small carcinoma lung cancer (NSCLC) patients. (a) Studies with immunotherapy in monotherapy, and (b) studies with immunotherapy combined with chemotherapy
**Figure S3**. Pooled analysis of progression‐free survival (PFS) for anti‐PD1 drugs, alone or in combination, in first‐line therapy for non‐small carcinoma lung cancer (NSCLC) patients. (a) Studies with immunotherapy in monotherapy, and (b) studies with immunotherapy combined with chemotherapy
**Figure S4**. Pooled analysis of progression‐free survival (PFS) for anti‐PD‐L1 drugs, alone or in combination, in first‐line therapy for non‐small carcinoma lung cancer (NSCLC) patients. (a) Studies with immunotherapy in monotherapy, and (b) studies with immunotherapy combined with chemotherapy
**Figure S5**. Pooled analysis of progression‐free survival (PFS) (a) and overall survival (OS) (b) for patients with tumors with PD‐L1 expression >50% treated with first‐line therapy for metastatic non‐small carcinoma lung cancer (NSCLC)
**Figure S6**. Pooled analysis of overall response rate (ORR) for anti‐PD1 drugs, alone or in combination, in first‐line therapy for non‐small carcinoma lung cancer (NSCLC) patients. (a) Studies with immunotherapy in monotherapy, and (b) studies with immunotherapy combined with chemotherapy
**Figure S7**. Pooled analysis of overall response rate (ORR) for anti‐PD‐L1 drugs, alone or in combination, in first‐line therapy for non‐small carcinoma lung cancer (NSCLC) patients. (a) Studies with immunotherapy in monotherapy and, (b) studies with immunotherapy combined with chemotherapy
**Figure S8**. Pooled analysis of grade 3–5 adverse events (AEs) for anti‐PD‐1 drugs, alone or in combination, in first‐line therapy for non‐small carcinoma lung cancer (NSCLC) patients. (a) Studies with immunotherapy in monotherapy, and (b) studies with immunotherapy combined with chemotherapy
**Figure S9**. Pooled analysis of grade 3–5 adverse events (AEs) for anti‐PD‐L1 drugs, alone or in combination, in first‐line therapy for non‐small carcinoma lung cancer (NSCLC) patients. (a) Studies with immunotherapy in monotherapy, and (b) studies with immunotherapy combined with chemotherapy
**Figure S10**. Indirect analysis statistical approach to compare overall survival (OS) between anti‐PD1 and anti‐PD‐L1 in (a) monotherapy, and (b) in combination with chemotherapy. In each line comparison (a) is the control group, (b) is the PD‐1 group, and (c) is the PD‐L1 group
**Figure S11**. Indirect analysis statistical approach to compare progression‐free survival (PFS) between anti‐PD1 and anti‐PD‐L1 in (a) monotherapy, and (b) in combination with chemotherapy. In each line comparison (a) is the control group, (b) is the PD‐1 group, and (c) is the PD‐L1 group
**Figure S12**. Indirect analysis statistical approach to compare overall response rate between anti‐PD1 and anti‐PD‐L1 in monotherapy (a) and in combination with chemotherapy (b). In each line of comparison (a) is the control group, (b) is the PD‐1 group, and (c) is the PD‐L1 group
**Figure S13**. Indirect analysis statistical approach to compare the rate of grade 3–5 AEs between anti‐PD1 and anti‐PD‐L1 in monotherapy (a) and in combination with chemotherapy (b). In each line of comparison (a) is the control group, (b) is the PD‐1 group, and (c) is the PD‐L1 group

**Table S1**. Supporting Information

## References

[1] Arbour KC , Riely GJ . Systemic therapy for locally advanced and metastatic non‐small cell lung cancer: a review. JAMA. 2019;322(8):764–74. 31454018 10.1001/jama.2019.11058

[2] Langer CJ , Gadgeel SM , Borghaei H , Papadimitrakopoulou VA , Patnaik A , Powell SF , et al. Carboplatin and pemetrexed with or without pembrolizumab for advanced, non‐squamous non‐small‐cell lung cancer: a randomised, phase 2 cohort of the open‐label KEYNOTE‐021 study. Lancet Oncol. 2016;17(11):1497–508. 27745820 10.1016/S1470-2045(16)30498-3 PMC6886237

[3] Reck M , Rodríguez‐Abreu D , Robinson AG , Hui R , Csőszi T , Fülöp A , et al. Pembrolizumab versus chemotherapy for PD‐L1‐positive non‐small‐cell lung cancer. N Engl J Med. 2016;375(19):1823–33. 27718847 10.1056/NEJMoa1606774

[4] Carbone DP , Reck M , Paz‐Ares L , Creelan B , Horn L , Steins M , et al. First‐line nivolumab in stage IV or recurrent non‐small‐cell lung cancer. N Engl J Med. 2017;376(25):2415–26. 28636851 10.1056/NEJMoa1613493 PMC6487310

[5] Barlesi F , Nishio M , Cobo M , Steele N , Paramonov V , Parente B , et al. IMpower132: efficacy of atezolizumab (atezo) + carboplatin (carbo)/cisplatin (cis) + pemetrexed (pem) as 1L treatment in key subgroups with stage IV non‐squamous non‐small cell lung cancer (NSCLC). European Society for Medical Oncology (ESMO) 2018 Congress; October 22, 2018; Munich, Germany 2018. http://bit.ly/2QD5DTD

[6] Gandhi L , Rodríguez‐Abreu D , Gadgeel S , Esteban E , Felip E , de Angelis F , et al. Pembrolizumab plus chemotherapy in metastatic non‐small‐cell lung cancer. N Engl J Med. 2018;378(22):2078–92. 29658856 10.1056/NEJMoa1801005

[7] Paz‐Ares L , Luft A , Vicente D , Tafreshi A , Gümüş M , Mazières J , et al. Pembrolizumab plus chemotherapy for squamous non‐small‐cell lung cancer. N Engl J Med. 2018;379(21):2040–51. 30280635 10.1056/NEJMoa1810865

[8] Socinski MA , Jotte RM , Cappuzzo F , Orlandi F , Stroyakovskiy D , Nogami N , et al. Atezolizumab for first‐line treatment of metastatic nonsquamous NSCLC. N Engl J Med. 2018;378(24):2288–301. 29863955 10.1056/NEJMoa1716948

[9] Hellmann MD , Paz‐Ares L , Bernabe Caro R , Zurawski B , Kim SW , Carcereny Costa E , et al. Nivolumab plus ipilimumab in advanced non‐small‐cell lung cancer. N Engl J Med. 2019;381(21):2020–31. 31562796 10.1056/NEJMoa1910231

[10] Mok TS , Wu Y , Kudaba I , Kowalski DM , Cho BC , Turna HZ , et al. Pembrolizumab versus chemotherapy for previously untreated, PD‐L1‐expressing, locally advanced or metastatic non‐small‐cell lung cancer (KEYNOTE‐042): a randomised, open‐label, controlled, phase 3 trial. Lancet. 2019;393(10183):1819–30. 30955977 10.1016/S0140-6736(18)32409-7

[11] West H , McCleod M , Hussein M , Morabito A , Rittmeyer A , Conter HJ , et al. Atezolizumab in combination with carboplatin plus nab‐paclitaxel chemotherapy compared with chemotherapy alone as first‐line treatment for metastatic non‐squamous non‐small‐cell lung cancer (IMpower130): a multicentre, randomised, open‐label, phase 3 trial. Lancet Oncol. 2019;20(7):924–37. 31122901 10.1016/S1470-2045(19)30167-6

[12] Jotte R , Cappuzzo F , Vynnychenko I , Stroyakovskiy D , Rodríguez‐Abreu D , Hussein M , et al. Atezolizumab in combination with carboplatin and nab‐paclitaxel in advanced squamous NSCLC (IMpower131): results from a randomized phase III trial. J Thorac Oncol. 2020;15(8):1351–60. 32302702 10.1016/j.jtho.2020.03.028

[13] Rizvi NA , Cho BC , Reinmuth N , Lee KH , Luft A , Ahn MJ , et al. Durvalumab with or without tremelimumab vs standard chemotherapy in first‐line treatment of metastatic non‐small cell lung cancer: the MYSTIC phase 3 randomized clinical trial. JAMA Oncol. 2020;6(5):661–74. 32271377 10.1001/jamaoncol.2020.0237 PMC7146551

[14] Herbst RS , Giaccone G , de Marinis F , Reinmuth N , Vergnenegre A , Barrios CH , et al. Atezolizumab for first‐line treatment of PD‐L1‐selected patients with NSCLC. N Engl J Med. 2020;383(14):1328–39. 32997907 10.1056/NEJMoa1917346

[15] Mavridis D , Salanti G . Exploring and accounting for publication bias in mental health: a brief overview of methods. Evid Based Ment Health. 2014;17:11–5. 24477532 10.1136/eb-2013-101700 PMC13404689

[16] Mavridis D , Salanti G . How to assess publication bias: funnel plot, trim‐and‐fill method and selection models. Evid Based Ment Health. 2014;17:30. 24477535 10.1136/eb-2013-101699 PMC13404685

[17] Catalá‐López F , Tobías A , Cameron C , Moher D , Hutton B . Network meta‐analysis for comparing treatment effects of multiple interventions: an introduction. Rheumatol Int. 2014;34(11):1489–96. 24691560 10.1007/s00296-014-2994-2

[18] Santos E , Ferreira RO , Marques A . How to perform and interpret a network meta‐analysis for indirect and mixed comparasions: key methodological strategies. Rev Enf Ref. 2016;8:133–40.

[19] Zinner RG , Obasaju CK , Spigel DR , Weaver RW , Beck JT , Waterhouse DM , et al. PRONOUNCE: randomized, open‐label, phase III study of first‐line pemetrexed + carboplatin followed by maintenance pemetrexed versus paclitaxel + carboplatin + bevacizumab followed by maintenance bevacizumab in patients ith advanced nonsquamous non‐small‐cell lung cancer. J Thorac Oncol. 2015;10(1):134–42. 25371077 10.1097/JTO.0000000000000366 PMC4276572

[20] Banna GL , Cantale O , Bersanelli M , Del Re M , Friedlaender A , Cortellini A , et al. Are anti‐PD1 and anti‐PD‐L1 alike? The non‐small‐cell lung cancer paradigm. Oncol Ver. 2020;14(2):490. 10.4081/oncol.2020.490 PMC7385529 32782728

[21] Enrico D , Paci A , Chaput N , Karamouza E , Besse B . Antidrug antibodies against immune checkpoint blockers: impairment of drug efficacy or indication of immune activation? Clin Cancer Res. 2020;26(4):787–92. 31757876 10.1158/1078-0432.CCR-19-2337

[22] Brahmer JR , Tykodi SS , Chow LQ , Hwu WJ , Topalian SL , Hwu P , et al. Safety and activity of anti‐PD‐L1 antibody in patients with advanced cancer. N Engl J Med. 2012;366(26):2455–65. 22658128 10.1056/NEJMoa1200694 PMC3563263

[23] Topalian SL , Hodi FS , Brahmer JR , Gettinger SN , Smith DC , McDermott DF , et al. Safety, activity, and immune correlates of anti‐PD‐1 antibody in cancer. N Engl J Med. 2012;366(26):2443–54. 22658127 10.1056/NEJMoa1200690 PMC3544539

[24] Duan J , Cui L , Zhao X , Bai H , Cai S , Wang G , et al. Use of immunotherapy with programmed cell death 1 vs programmed cell death ligand 1 inhibitors in patients with cancer: a systematic review and meta‐analysis. JAMA Oncol. 2020;6(3):375–84. 31876895 10.1001/jamaoncol.2019.5367 PMC6990765

[25] You W , Liu M , Miao J , Liao YQ , Song YB , Cai DK , et al. A network meta‐analysis comparing the efficacy and safety of anti‐PD‐1 with anti‐PD‐L1 in non‐small cell lung cancer. J Cancer. 2018;9(7):1200–6. 29675101 10.7150/jca.22361 PMC5907668

[26] Tartarone A , Roviello G , Lerose R , Roudi R , Aieta M , Zoppoli P . Anti‐PD‐1 versus anti‐PD‐L1 therapy in patients with pretreated advanced non‐small‐cell lung cancer: a meta‐analysis. Future Oncol. 2019;15(20):2423–33. 31237152 10.2217/fon-2018-0868

[27] Pillai RN , Behera M , Owonikoko TK , Kamphorst AO , Pakkala S , Belani CP , et al. Comparison of the toxicity profile of PD‐1 versus PD‐L1 inhibitors in non‐small cell lung cancer: a systematic analysis of the literature. Cancer. 2018;124(2):271–7. 28960263 10.1002/cncr.31043 PMC5761314

[28] Latchman Y , Wood CR , Chernova T , Chaudhary D , Borde M , Chernova I , et al. PD‐L2 is a second ligand for PD‐1 and inhibits T cell activation. Nat Immunol. 2001;2(3):261–8. 11224527 10.1038/85330

[29] Schmid P , Hegde PS , Zou W , Kowanetz M , Mariathasan S , Molinero L , et al. Association of PD‐L2 expression in human tumors with atezolizumab activity. J Clin Oncol. 2016;34(15):11506.

[30] Yearley JH , Gibson C , Yu N , Moon C , Murphy E , Juco J , et al. PD‐L2 expression in human tumors: relevance to anti‐PD‐1 therapy in cancer. Clin Cancer Res. 2017;23(12):3158–67. 28619999 10.1158/1078-0432.CCR-16-1761

