# Supplementary material for: Anti‐PD1 versus anti‐PD‐L1 immunotherapy in first‐line therapy for advanced non‐small cell lung cancer: A systematic review and meta‐analysis
Source: Thorac Cancer. 2021 Feb 14;12(7):1058–66. doi: 10.1111/1759-7714.13867 (PMC8017262; doi:10.1111/1759-7714.13867)
Supplement: Supplementary file 1 — Figure S1. Pooled analysis of overall survival (OS) for anti‐PD1 drugs, alone or in combination, in first‐line therapy for non‐small carcinoma lung cancer (NSCLC) patients. (a) Studies with immunotherapy in monotherapy, and (b) studies with immunotherapy combined with chemotherapy Figure S2. Pooled analysis of overall survival (OS) for anti‐PD‐L1drugs, alone or in combination, in first‐line therapy for non‐small carcinoma lung cancer (NSCLC) patients. (a) Studies with immunotherapy in monotherapy, and (b) studies with immunotherapy combined with chemotherapy Figure S3. Pooled analysis of progression‐free survival (PFS) for anti‐PD1 drugs, alone or in combination, in first‐line therapy for non‐small carcinoma lung cancer (NSCLC) patients. (a) Studies with immunotherapy in monotherapy, and (b) studies with immunotherapy combined with chemotherapy Figure S4. Pooled analysis of progression‐free survival (PFS) for anti‐PD‐L1 drugs, alone or in combination, in first‐line therapy for non‐small carcinoma lung cancer (NSCLC) patients. (a) Studies with immunotherapy in monotherapy, and (b) studies with immunotherapy combined with chemotherapy Figure S5. Pooled analysis of progression‐free survival (PFS) (a) and overall survival (OS) (b) for patients with tumors with PD‐L1 expression >50% treated with first‐line therapy for metastatic non‐small carcinoma lung cancer (NSCLC) Figure S6. Pooled analysis of overall response rate (ORR) for anti‐PD1 drugs, alone or in combination, in first‐line therapy for non‐small carcinoma lung cancer (NSCLC) patients. (a) Studies with immunotherapy in monotherapy, and (b) studies with immunotherapy combined with chemotherapy Figure S7. Pooled analysis of overall response rate (ORR) for anti‐PD‐L1 drugs, alone or in combination, in first‐line therapy for non‐small carcinoma lung cancer (NSCLC) patients. (a) Studies with immunotherapy in monotherapy and, (b) studies with immunotherapy combined with chemotherapy Figure S8. Pooled analysis of grade [file TCA-12-1058-s001.docx]

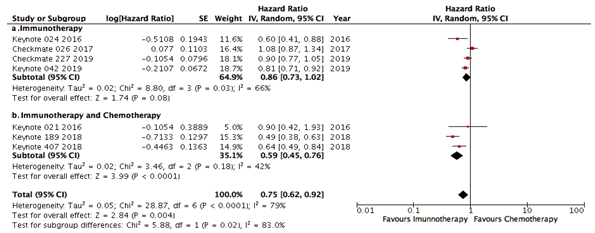


Supplemental Figure 1. Pooled analysis of overall survival for anti-PD1drugs, alone or in combination, in first line therapy for non-small carcinoma lung cancer patients. In (a) studies with immunotherapy in monotherapy and in (b) studies with immunotherapy combined with chemotherapy.


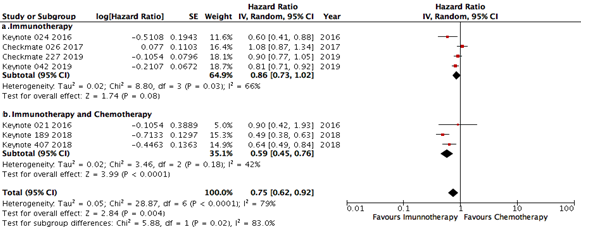


Supplemental Figure 2. Pooled analysis of overall survival for anti-PD-L1drugs, alone or in combination, in first line therapy for non-small carcinoma lung cancer patients. In (a) studies with immunotherapy in monotherapy and in (b) studies with immunotherapy combined with chemotherapy.


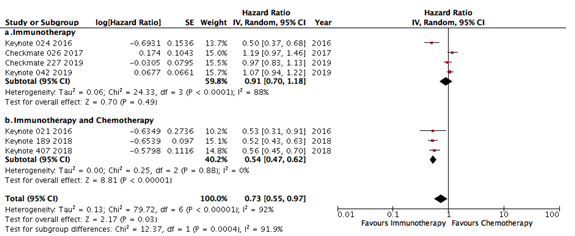


Supplemental Figure 3. Pooled analysis of progression free survival for anti-PD1 drugs, alone or in combination, in first line therapy for non-small carcinoma lung cancer patients. In (a) studies with immunotherapy in monotherapy and in (b) studies with immunotherapy combined with chemotherapy.


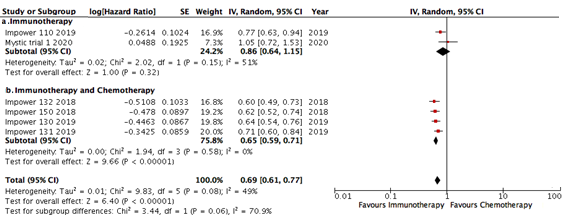


Supplemental Figure 4. Pooled analysis of progression free survival for anti-PD-L1 drugs, alone or in combination, in first line therapy for non-small carcinoma lung cancer patients. In (a) studies with immunotherapy in monotherapy and in (b) studies with immunotherapy combined with chemotherapy.


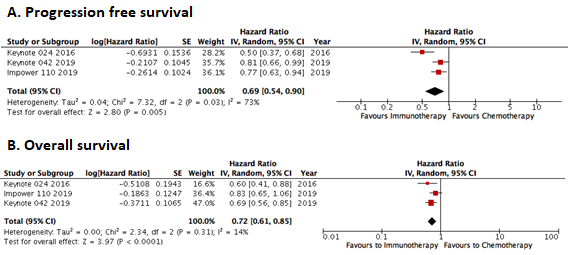


Supplemental Figure 5. Pooled analysis of progression free survival (A) and overall survival (B) for patients with tumors with PD-L1 expression>50% treated in first line therapy for metastatic non-small carcinoma lung cancer.


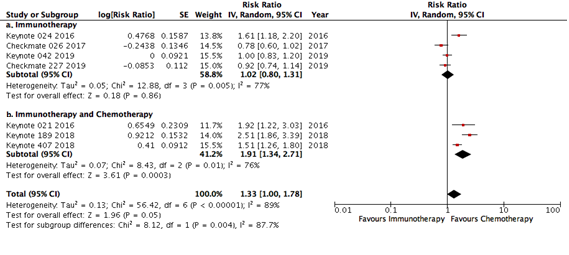


Supplemental Figure 6. Pooled analysis of overall response rate, for anti-PD1 drugs, alone or in combination, in first line therapy for non-small carcinoma lung cancer patients. In (a) studies with immunotherapy in monotherapy and in (b) studies with immunotherapy combined with chemotherapy.


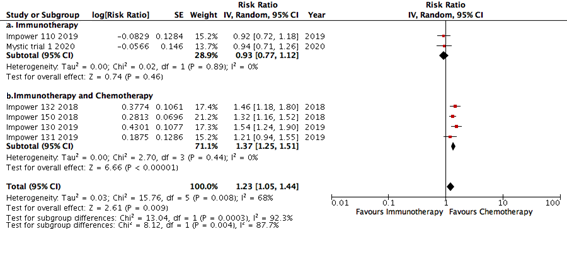


Supplemental Figure 7. Pooled analysis of overall response rate, for anti-PD-L1 drugs, alone or in combination, in first line therapy for non-small carcinoma lung cancer patients. In (a) studies with immunotherapy in monotherapy and in (b) studies with immunotherapy combined with chemotherapy


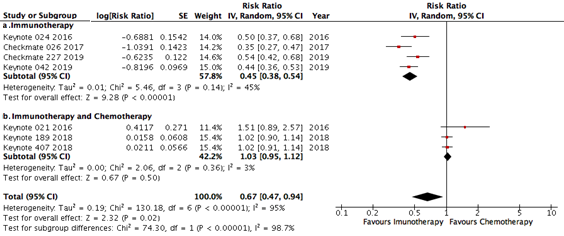


Supplemental Figure 8. Pooled analysis of grade 3-5 AEs for anti-PD-1 drugs, alone or in combination, in first line therapy for non-small carcinoma lung cancer patients. In (a) studies with immunotherapy in monotherapy and in (b) studies with immunotherapy combined with chemotherapy


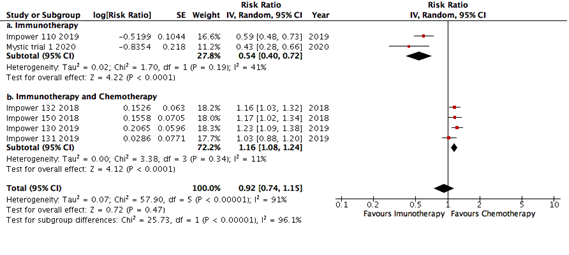


Supplemental Figure 9. Pooled analysis of grade 3-5 AEs for anti-PD-L1 drugs, alone or in combination, in first line therapy for non-small carcinoma lung cancer patients. In (a) studies with immunotherapy in monotherapy and in (b) studies with immunotherapy combined with chemotherapy


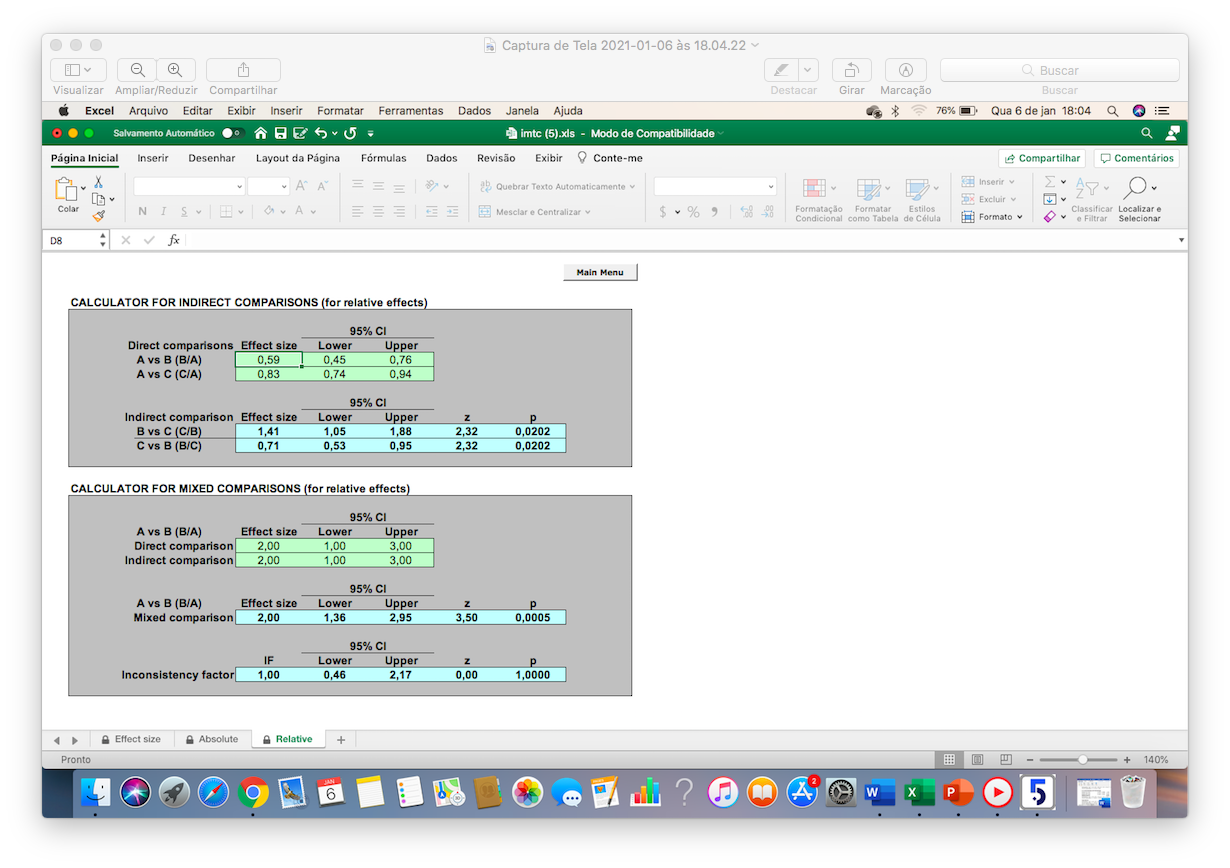


Supplemental Figure 10. Indirect analysis statistical approach to compare overall survival between anti-PD1 and anti-PD-L1 in monotherapy (upper image - A) and in combination with chemotherapy (below image - B). In each line of the comparisons, A is the control group, B is the PD-1 group and C is the PD-L1 group.


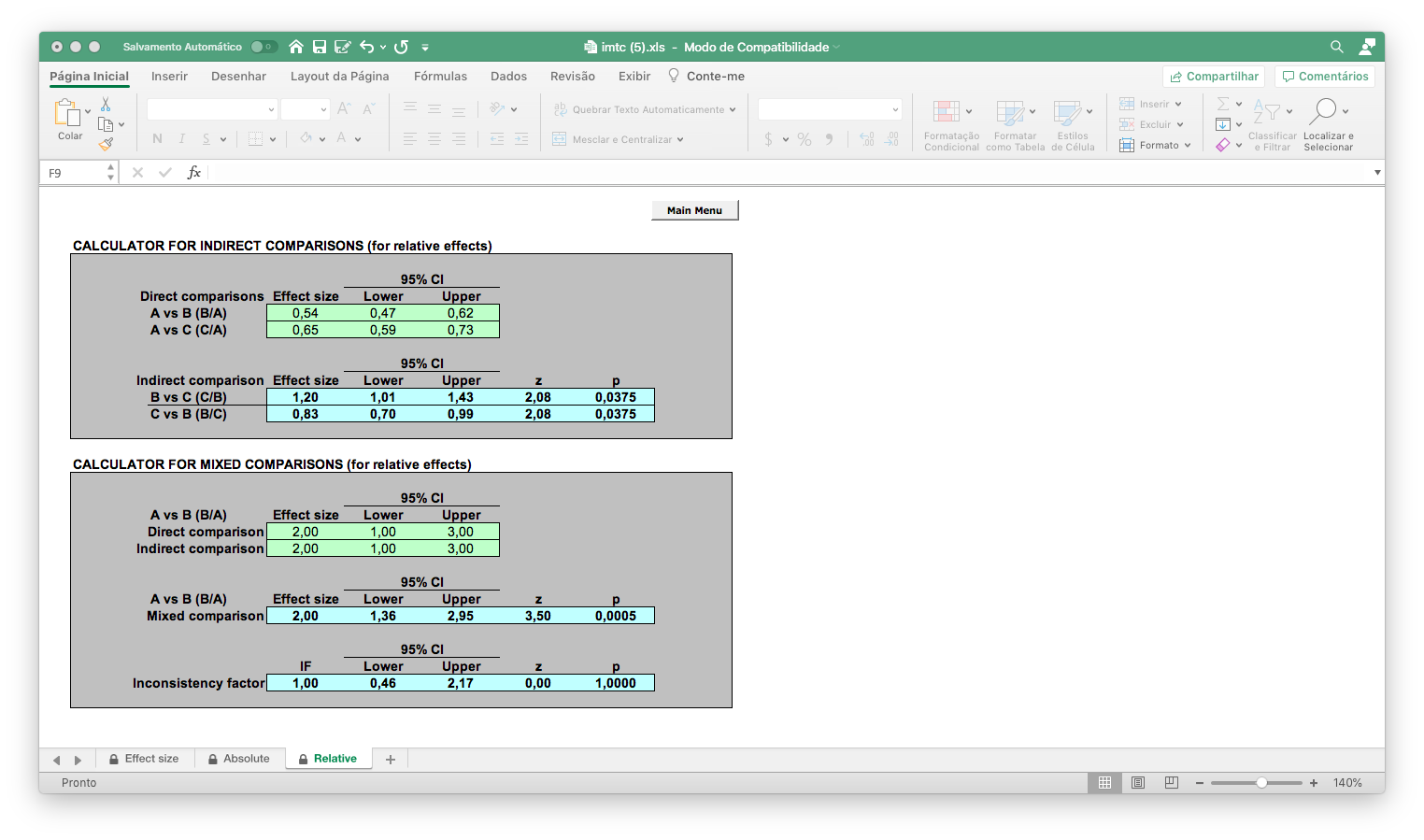


Supplemental Figure 11. Indirect analysis statistical approach to compare progression free survival between anti-PD1 and anti-PD-L1 in monotherapy (upper image - A) and in combination with chemotherapy (below image - B). In each line of the comparisons, A is the control group, B is the PD-1 group and C is the PD-L1 group.


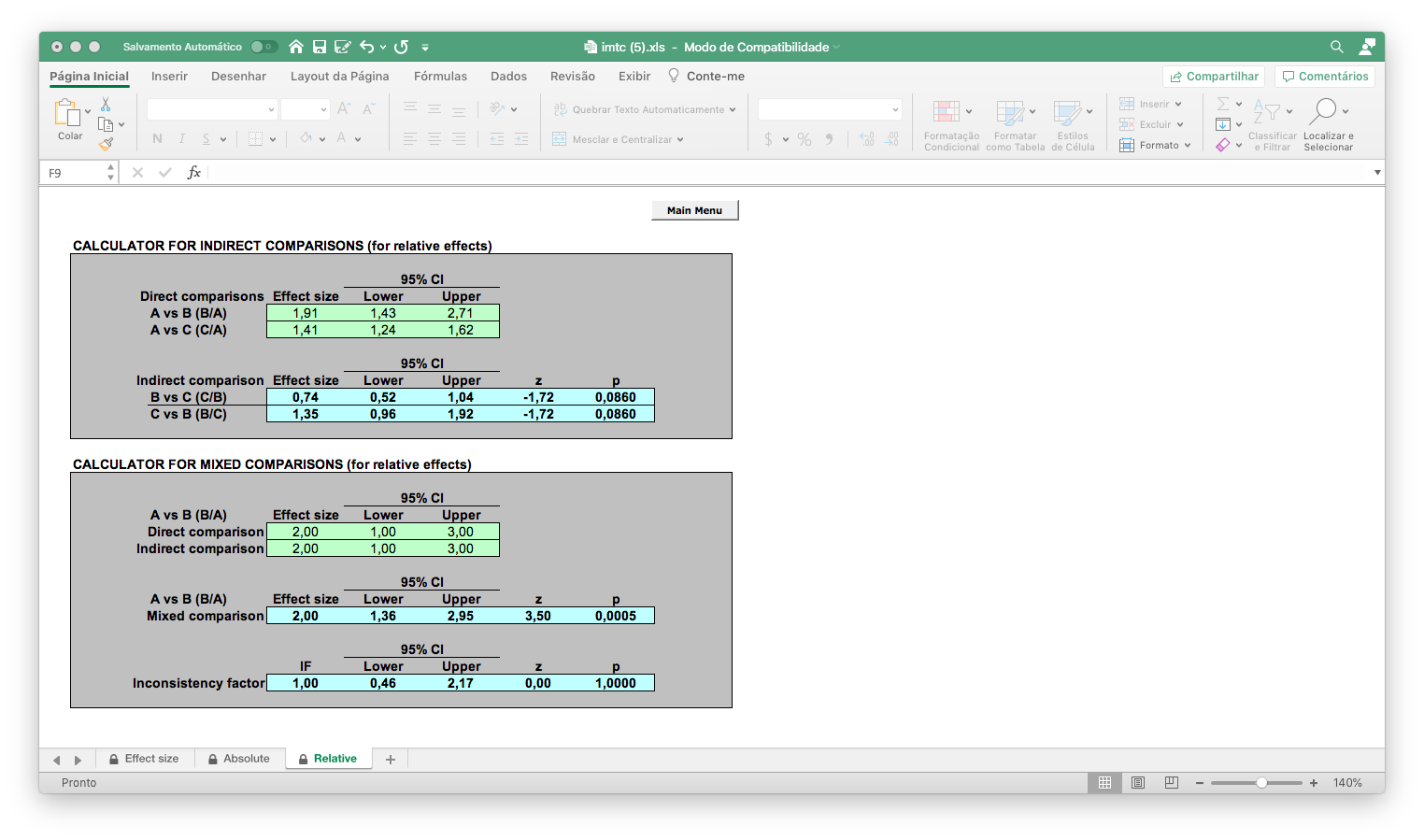


Supplemental Figure 12. Indirect analysis statistical approach to compare overall response ratel between anti-PD1 and anti-PD-L1 in monotherapy (upper image - A) and in combination with chemotherapy (below image - B). In each line of the comparisons, A is the control group, B is the PD-1 group and C is the PD-L1 group.


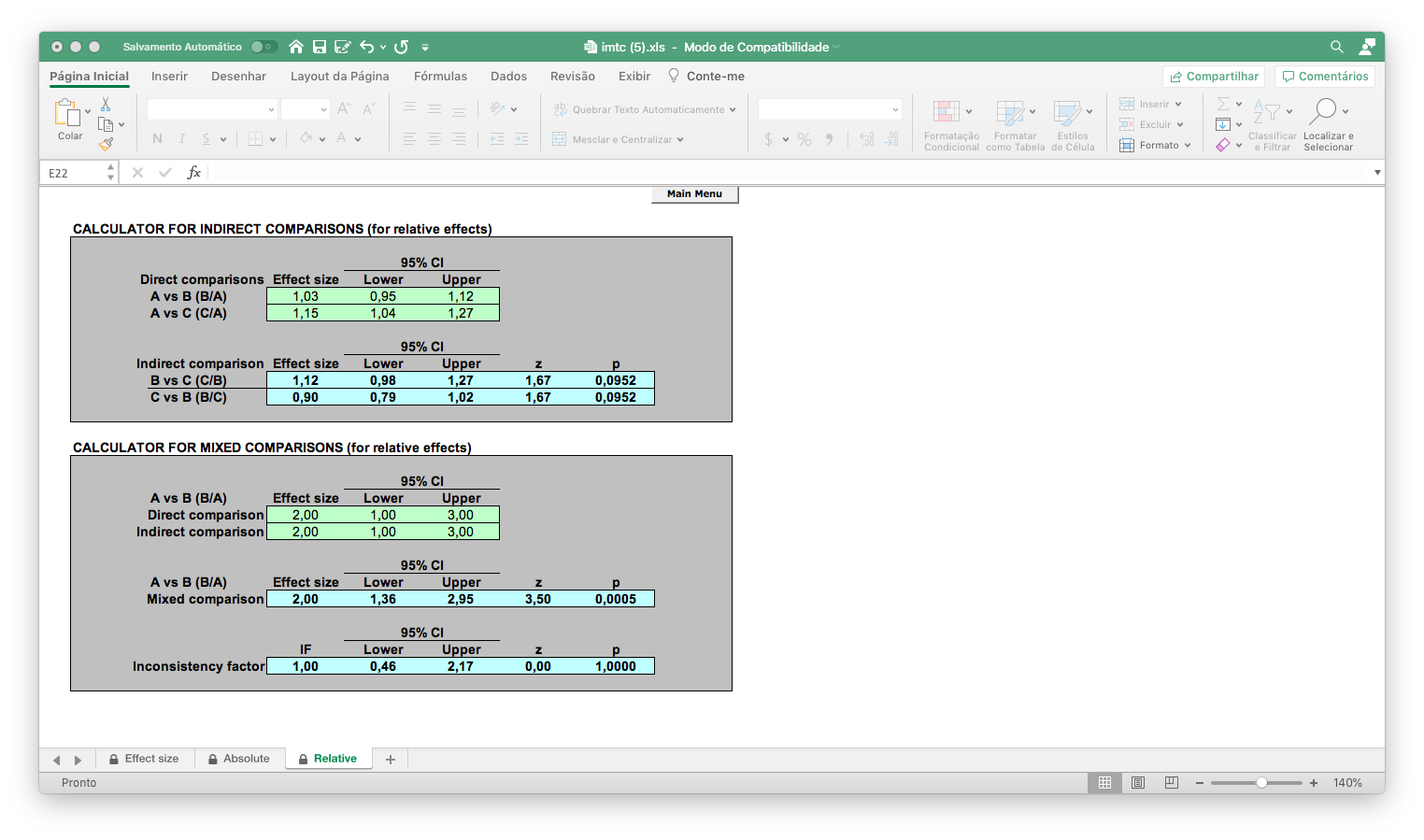


Supplemental Figure 13. Indirect analysis statistical approach to compare the rate of grade 3-5 AEs between anti-PD1 and anti-PD-L1 in monotherapy (upper image - A) and in combination with chemotherapy (below image - B). In each line of the comparisons, A is the control group, B is the PD-1 group and C is the PD-L1 group.
