# Supplementary material for: Anti‐PD1 versus anti‐PD‐L1 immunotherapy in first‐line therapy for advanced non‐small cell lung cancer: A systematic review and meta‐analysis
Source: Thorac Cancer. 2021 Feb 14;12(7):1058–66. doi: 10.1111/1759-7714.13867 (PMC8017262; doi:10.1111/1759-7714.13867)
Supplement: Supplementary file 2 — Table S1. Supporting Information [file TCA-12-1058-s002.docx]

| **Study** | **Histology in intervention** | **Histology in control** | **Treatment protocol** | **ECOG 0 in intervention** | **ECOG 0 in control** | **Current or former smoke in intervention** | **Current or former smoke in control** | **PDL1 expression** | **PDL1 antibody** |
| --- | --- | --- | --- | --- | --- | --- | --- | --- | --- |
| KEYNOTE 024 | 18.8% squamous / 81.2% non-squamous | 17.9% squamous / 82.1% non-squamous | Platinum * +- Pembrolizumab | 35.1 (%) | 35.1 (%) | 96.8 (%) | 87.4 (%) | ≥50% | 22C3 pharmDx |
| CHECKMATE 026 | 24.0% squamous / 76.0% non-squamous | 24.0% squamous / 76.0% non-squamous | Platinum * +- Nivolumab | 31.0 (%) | 34.0 (%) | 88.0 (%) | 87.0 (%) | ≥1% | anti–PD-L1 28-8 |
| CHECKMATE 227 | 29.5% squamous / 70.5% non-squamous | 29.2% squamous / 70.8% non-squamous | Platinum * +- Nivolumab | 35.9 (%) | 33.8 (%) | 86.4 (%) | 85.6 (%) | ≥1% | anti–PD-L1 28-8 |
| KEYNOTE 042 | 38.0% squamous / 62.0% non-squamous | 39.0% squamous / 61.0% non-squamous | Carboplatin + paclitaxel or pemetrexed +- Pembrolizumab | 31.0 (%) | 30.0 (%) | 78.0 (%) | 78.0 (%) | ≥1% | 22C3 pharmDx |
| MYSTIC | 31.9% squamous / 68.1% non-squamous | 32.5% squamous / 67.5% non-squamous | Platinum * +- Durvalumab | 35.0 (%) | 43.2 (%) | 85.3 (%) | 84.7 (%) | ≥1% | Ventana PD-L1 SP263 |
| IMPOWER 110 | 30.7% squamous / 69.3% non-squamous | 30.3% squamous / 69.7% non-squamous | Platinum + Pemetrexed/Gemcitabine +/- Atezolizumab | 35.0 (%) | 36.8 (%) | 86.6 (%) | 87.4 (%) | ≥1% | Ventana SP142 |
|  |  |  |  |  |  |  |  |  |  |
| KEYNOTE 021 | 100% Non-squamous | 100% Non-squamous | Carboplatin + Pemetrexede +/- Pembrolizumab | 40.0 (%) | 46.0 (%) | 75.0 (%) | 86.0 (%) | All | 22C3 pharmDx |
| IMPOWER 132 | 100% Non-squamous | 100% Non-squamous | Platin + pemetrexede +/- Atezolizumab | 43.2 (%) | 40.1 (%) | 87.3 (%) | 87.4(%) | All | Ventana SP142 |
| KEYNOTE 189 | 100% Non-squamous | 100% Non-squamous | Carboplatin + Pemetrexede +/- Pembrolizumab | 45.4 (%) | 38.8 (%) | 88.3 (%) | 87.9 (%) | All | 22C3 pharmDx |
| KEYNOTE 407 | 100% Squamous | 100% Squamous | Carboplatin + Taxol +/- Pembrolizumab | 26.3 (%) | 32.0 (%) | 92.1 (%) | 93.2 (%) | All | 22C3 pharmDx |
| IMPOWER 150 | 100% Non-squamous | 100% Non-squamous | Carboplatin + nab-paclitaxel + Bevacizumab +/- Atezolizumab | 40.1 (%) | 45.1 (%) | 79.5 (%) | 81.8 (%) | All | Ventana SP142 |
| IMPOWER 130 | 100% Non-squamous | 100% Non-squamous | Carboplatin + nab-paclitaxel +/- Atezolizumab | 42.0 (%) | 40.0 (%) | 89.0 (%) | 92.0 (%) | All | Ventana SP142 |
| IMPOWER 131 | 100% Squamous | 100% Squamous | Carboplatin + nab-paclitaxel +/- Atezolizumab | 32.8 (%) | 32.4 (%) | 90.9 (%) | 92.9 (%) | All | Ventana SP142 |

* Investigator’s choice of platinum-based chemotherapy: carboplatin plus pemetrexed, cisplatin plus pemetrexed, carboplatin plus gemcitabine, cisplatin plus gemcitabine, or carboplatin plus paclitaxel
